# Temporal and spatial regulation of translation in the mammalian oocyte via the mTOR–eIF4F pathway

**DOI:** 10.1038/ncomms7078

**Published:** 2015-01-28

**Authors:** Andrej Susor, Denisa Jansova, Renata Cerna, Anna Danylevska, Martin Anger, Tereza Toralova, Radek Malik, Jaroslava Supolikova, Matthew S. Cook, Jeong Su Oh, Michal Kubelka

**Affiliations:** 1 Institute of Animal Physiology and Genetics, ASCR, Rumburska 89, 277 21 Libechov, Czech Republic; 2 CEITEC-Veterinary Research Institute, Hudcova 296/70, 621 00 Brno, Czech Republic; 3 Institute of Molecular Genetics, ASCR, Videnska 1083, 142 20 Prague, Czech Republic; 4 The Eli and Edythe Broad Center of Regeneration Medicine and Stem Cell Research, UCSF, San Francisco, California 94143, USA; 5 Department of Genetic Engineering, Sungkyunkwan University, Gyeonggi-do, Suwon 440-746, South Korea

## Abstract

The fully grown mammalian oocyte is transcriptionally quiescent and utilizes only transcripts synthesized and stored during early development. However, we find that an abundant RNA population is retained in the oocyte nucleus and contains specific mRNAs important for meiotic progression. Here we show that during the first meiotic division, shortly after nuclear envelope breakdown, translational hotspots develop in the chromosomal area and in a region that was previously surrounded the nucleus. These distinct translational hotspots are separated by endoplasmic reticulum and Lamin, and disappear following polar body extrusion. Chromosomal translational hotspots are controlled by the activity of the mTOR–eIF4F pathway. Here we reveal a mechanism that—following the resumption of meiosis—controls the temporal and spatial translation of a specific set of transcripts required for normal spindle assembly, chromosome alignment and segregation.

## Abstract (web summary)

Meiotic maturation of oocytes and early development of mammalian embryos is largely dependent on the translation of mRNAs stored in the oocyte. Here the authors uncover a population of mRNA retained in the oocyte nucleus whose translation is spatially and temporally regulated by the mTOR–eIF4F pathway during meiosis.

## Graphical abstract

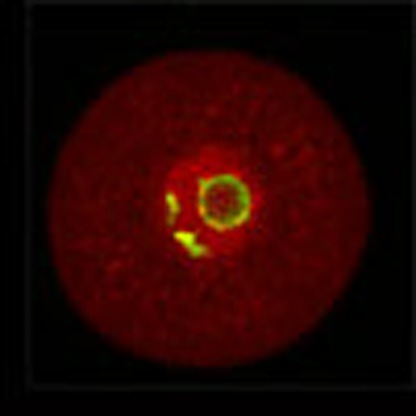

Post-transcriptional control of gene expression at the level of translation has emerged as an important cellular function in normal development^1^. A hallmark of early development in mammals is the reliance on translation and utilization of stored RNA and proteins rather than *de novo* transcription of genes to sustain rapid development^1^,^2^,^3^. After a period of active transcription during growth, the nucleus (germinal vesicle, GV) of mammalian oocytes becomes transcriptionally inactive^4^. In the absence of transcription, the completion of meiosis and early embryo development in mammals relies significantly on maternally synthesized RNAs^1^,^5^,^6^. Therefore, regulation of gene expression in oocytes is controlled almost exclusively at the level of mRNA stabilization and translation. At the onset of the first meiotic division, nuclear envelope breakdown (NEBD) occurs, chromosomes condense and a bipolar spindle is formed from the microtubule organizing centres^7^. During meiosis I, the spindle migrates from the centre of the oocyte to the cortex, and the oocyte undergoes an asymmetric division resulting in a large egg competent for fertilization and a relatively small polar body. Proper positioning of the spindle during asymmetric cell division ensures correct partitioning of cellular determinants^8^. How these events are orchestrated remains unclear.

The early development of all animals is programmed by maternal RNAs and proteins deposited in the egg^1^. The localization of mRNA within a cell is an essential prerequisite for the correct propagation of genetic information and it is also a very efficient way to orchestrate cellular processes. In many species, including *Drosophila and Xenopus*, the synthesis of proteins is localized by compartmentalization of mRNAs^9^,^10^,^11^. This is critical for the determination of the animal and vegetal poles of *Xenopus* embryos, which requires accurate asymmetric distribution of several mRNAs^12^. However, little is known about the patterning of mammalian oocytes through localization of mRNAs, except for reported accumulation of RNA in the cortex of the oocyte^13^,^14^.

Control of cap-dependent translation occurs mainly at the initiation step through the regulation of activity of the cap-binding protein complex eIF4F. This complex consists of three subunits: eIF4E, which specifically recognizes the cap structure, eIF4A helicase, and a bridging protein, eIF4G, responsible for eIF4F complex integrity^15^. The most important factor is probably the cap-binding protein, eIF4E. Its binding capacity is believed to be enhanced by the phosphorylation on S209, which correlates with an increase in translation^16^,^17^,^18^. EIF4E participates in the formation of the eIF4F complex, and it is also controlled via the regulatory proteins binding to eIF4E, the 4E-binding proteins (4E-BPs), which have to undergo phosphorylation to dissociate from eIF4E in such a way to enable its coupling with eIF4G and formation of the functional eIF4F complex^19^. EIF4E also stimulates eIF4A helicase activity^20^, which is important for unwinding the mRNAs with long and highly structured 5′UTRs that have been previously reported to be translated in an eIF4E-dependent manner^21^. The kinase responsible for phosphorylating 4E-BPs on several sites is mTOR, which itself is regulated by the PI3K/Akt signalling pathway^18^. Two different mTOR complexes have been described that are associated with two different regulatory proteins, raptor and rictor. mTORC1 represents the complex of mTOR with raptor that is sensitive to rapamycin (Rap) and is responsible for 4E-BP1 and ribosomal protein S6 kinase (S6K) phosphorylation. Alternatively, mTORC2, the Rap-resistant mTOR–rictor complex, regulates cytoskeletal changes and Akt kinase phosphorylation^22^. Although Cdk1 kinase has been shown to phosphorylate 4E-BP1 on S65 and T70^23^ and Plk1 seems to be responsible for the phosphorylation on S112^24^, phosphorylation of these sites requires priming phosphorylation on T37 and T46, which is mediated by mTOR^19^. Increased phosphorylation of 4E-BP1 has also been shown during meiotic progression of mammalian oocytes^25^,^26^, and recently different phosphorylated forms of 4E-BP1 have been shown to co-localize with the meiotic spindle in mouse oocytes^27^. In conclusion, mTOR appears to be of crucial importance for the formation of the active eIF4F complex, which stimulates the translation of eIF4E-sensitive mRNAs characterized by a 5′ terminal oligopyrimidine (TOP) motif^28^.

We have used a molecular and biochemical approach to identify the previously uncharacterized *in situ* translation in mammalian oocytes. We show a direct link between localization of an enriched population of poly(A)-RNAs and active translation, as well as of active components of the mTOR–eIF4F regulatory pathway in the newly described and distinctly bordered areas around the chromosomes and spindle. They form shortly after NEBD and are likely to contribute to spindle formation as well as the fidelity of chromosome segregation. Together these findings suggest a spatiotemporally regulated translational control of chromosome segregation and functional spindle formation mediated by mTOR–eIF4F during meiotic progression of mammalian oocytes.

## Results

### Cap-dependent translation is essential for genomic stability

**Figure 1:**
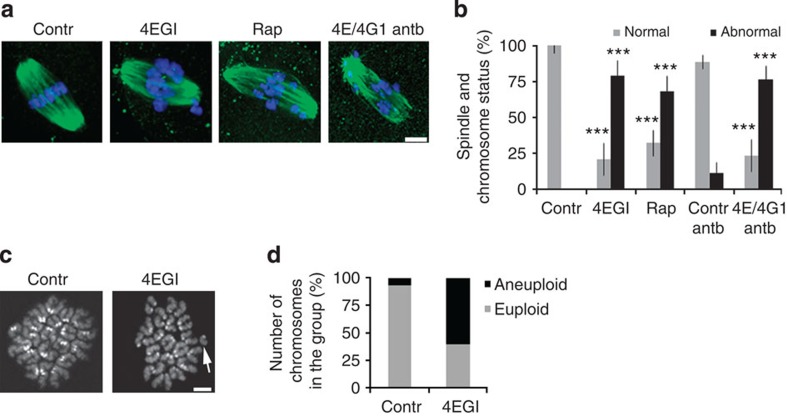
Disruption of the mTOR–eIF4F pathway affects genomic stability in meiosis I and impairs translation of specific mRNAs. (**a**,**b**) Oocytes treated with 4EGI or Rap or microinjected with an eIF4E/eIF4G1 antibody cocktail show aberrant spindle formation. Data are represented as the mean±s.d. Asterisks denote *P*<0.001; NS, non significant; according to a Student’s *t*-test; *n*≥35. Tubulin (green) and DAPI (blue). Scale bar, 5 μm. (**c**,**d**) Chromosomal spreads show aneuploidy and loose chromosomes/chromatids in oocytes with a downregulated 4F complex. Representative image from two independent experiments is shown (*n*≥14); arrow denotes separated chromatid. Scale bar, 10 μm. See also Supplementary Figs 1–3.

Cap-dependent translation is known to be important during the G1/S transition in somatic cells, and it has also been shown to be involved in the regulation of meiotic progression in mammalian oocytes. The overall translation gradually decreases during oocyte meiotic maturation, but the activators of cap-dependent translation become activated during this period, implying a role for translation of specific mRNAs to regulate meiosis^25^,^26^. Here we show that the downregulation of mTOR and the supression of the formation of the eIF4F complex^28^ (which is involved in the cap-dependent translation Supplementary Fig. 1a,b) in maturing mouse oocytes using a specific inhibitor of interaction between eIF4E and eIF4G1, 4EGI-1 (ref. 29) (4EGI), leads to 79% (*P*<0.001) of oocytes with significant defects in chromosome alignment and spindle morphology in metaphase I and II (Fig. 1a,b and Supplementary Fig. 2a,b,e), without blocking meiotic progression *per se* (Supplementary Fig. 2c,d). This in turn results in chromosome aneuploidy. Indeed, chromosomal spreads of inhibitor-treated oocytes revealed a 60% aneuploidy rate in MII oocytes (Fig. 1c,d).

Similar results were obtained using eIF4E (4E) or eIF4G1 (4G1) antibodies, as well as (Rap, an inhibitor of mTOR. Although the oocytes extruded a polar body and appeared normal (Supplementary Fig. 2d), abnormalities in spindle assembly and chromosome alignment were observed (Fig. 1a,b and Supplementary Fig. 2a,b,e). This phenotype was observed when oocytes were cultured in the presence of 4EGI (79%; *P*<0.001), Rap (68%; *P*<0.001) or microinjected with antibodies against eIF4E and eIF4G1 (76.5%; *P*<0.001). When global translation was disrupted by puromycin, oocytes progressed through metaphase I stage; however, cytokinesis was impaired and polar body extrusion did not occur^30^. Both 4EGI- and Rap-treated oocytes show no change in eIF2a phosphorylation (Supplementary Fig. 3), suggesting that such treatments do not induce a translational stress response^31^. Oocytes with a disrupted mTOR–eIF4F pathway are able to progress through meiosis I and extrude a first polar body, however, severe errors in chromosome segregation occur.

### The mTOR/4F axis is highly active at the onset of meiosis

The mTOR–eIF4F pathway is responsible for the early recognition of capped mRNAs during translation initiation, and this interaction is stabilized by eIF4G1 resulting in the activation of translation initiation. Interaction between eIF4E and eIF4G1 is mainly regulated by mTOR-mediated phosphorylation of 4E-BP1 (refs 19, 32).

**Figure 2:**
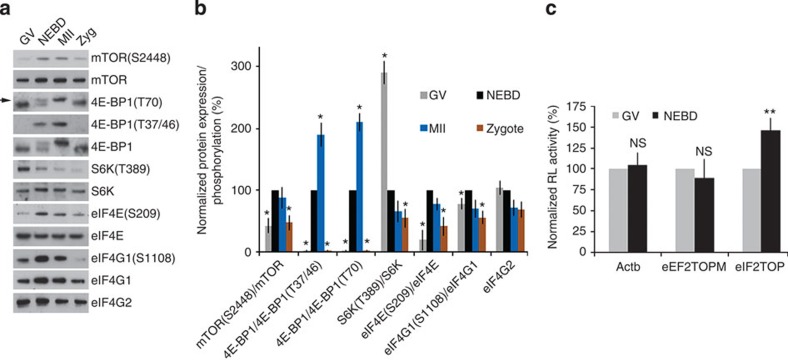
The mTOR–eIF4F translational pathway is highly active at the onset of meiosis and downregulated after fertilization. (**a**,**b**) Immunoblot analysis of the key players of the mTOR–eIF4F pathway shows their upregulation after NEBD (3 h PIW). Ratios of the abundance of the phosphorylated form of mTOR, 4E-BP1, S6K, eIF4E end eIF4G1 are presented in the form of a bar chart. Data are represented as the mean±s.d.; values obtained for NEBD stage were set as 100%; asterisk denotes statistically significant differences (Student’s *t*-test: *P*<0.05); *n*≥3; arrow denotes phospho-4E-BP1(T70). See also Supplementary Fig. 6. (**c**) RNA RL construct with TOP motif (eEF2^TOP^) has increased translation after NEBD. Data are represented as the mean±s.d. **P<0.01, according to a Student’s *t*-test. Data are representative of at least three independent experiments. Values obtained for GV stage were set as 100%. See also Supplementary Fig. 10.

To better understand the observed phenotype of cap-dependent translational regulation we decided to perform a detailed analysis of the expression, localization, and activation of the mTOR and 4F pathway components. Our data show that the mTOR and 4F pathways become activated shortly (3 h post IBMX wash; PIW) after NEBD (Fig. 2a,b). We detected increased expression as well as phosphorylation-dependent activation of mTOR (Fig. 2a,b) with parallel the phosphorylation of its target substrate, 4E-BP1 (Fig. 2a,b). Similarly, substantial increase in eIF4E phosphorylation accompanied by increased expression levels and phosphorylation of eIF4G1 was observed after NEBD (Fig. 2a,b). These two proteins belong to the key translational factors that promote translation of specific mRNAs^28^,^33^. On the other hand, another mTOR substrate, S6K, which was shown previously to be involved in the regulation of proteosynthesis^34^, became gradually dephosphorylated after NEBD (Fig. 2a,b). It should be noted that the expression level of the non-cap-dependent translation promoter^35^,^36^ eIF4G2 was constant or even slightly decreased during oocyte maturation (Fig. 2a,b). The data suggest that the critical period for mTOR–eIF4F translational pathway activation is the time at or shortly after NEBD, with activation being maintained up to the MII stage. The translational complex becomes remodelled/deactivated after fertilization with parallel dephosphorylation of 4E-BP1 and eIF4E (Fig. 2a,b and Supplementary Fig. 10).

We next tested whether the activation of the mTOR–eIF4F pathway regulates translation of injected renilla luciferase (RL) reporters. Because it is known that the eIF4F complex promotes the translation of TOP RNAs^28^, we microinjected the oocytes with reporter RNA: RL constructs containing an upstream non-TOP sequence (Actb), a mutated oligopirimidine sequence (eEF2^TOPM^), or a canonical oligopirimidine sequence (eEF2^TOP^). Firefly luciferase (FL) was used as a microinjection control. Oocytes injected with the reporter containing a canonical TOP sequence showed a 46% increase in RL signal (*P*<0.01) after NEBD. On the other hand, its translation was low before NEBD in the GV oocyte. The translation of the other reporters containing either non-TOP or mutated TOP sequences was unaffected after NEBD (Fig. 2c). These data suggest that the mTOR–eIF4F pathway becomes highly activated after NEBD and regulates mRNAs with TOP sequences.

### *In situ* translation reveals two distinct hotspots after NEBD

**Figure 3:**
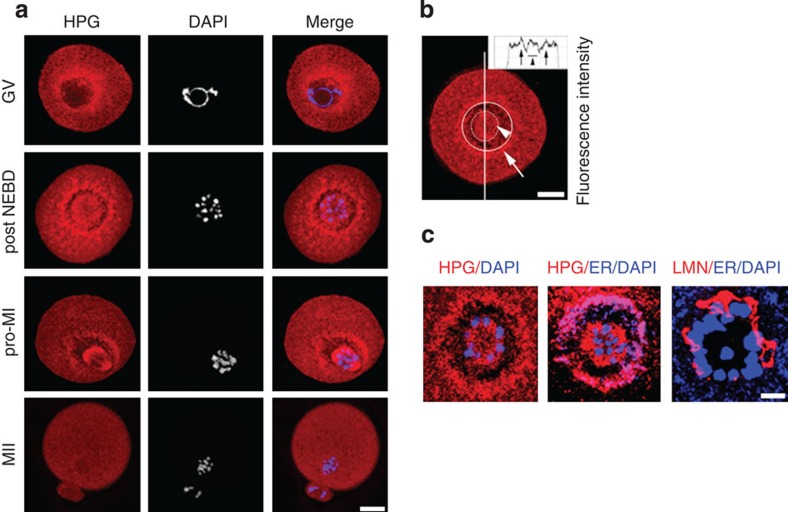
*In situ* translation shows two distinct hotspots in oocytes. (**a**) Oocytes in different stages were cultured in the presence of HPG for 30 min. HPG (red); DAPI (blue). (**b**) NEBD oocytes cultured for 30 min in HPG. Histogram shows HPG intensity depicted along the white line. The arrowhead and arrow indicates the CTA (dot line) and PTA (uninterupted line), respectively; HPG (red). See also Supplementary Movie 1. (**c**) ER tracker shows perispindular localization of ER and overlaps with the PTA post NEBD. Polymerized LMN separates the CTA from the PTA. HPG, LMN, (red); ER tracker and DAPI (blue). Data are representative of at least two independent experiments; scale bar, ~10 μm.

Using the methionine analogue homopropargylglycine (HPG; L-homopropargylglycine)^37^ we analysed nascent proteosynthesis in the oocyte. Oocytes were exposed to HPG for a short cultivation period^36^ (30 min), which facilitated incorporation into translated proteins and subsequent visualization using confocal microscopy. Our results showed that although the whole oocyte was translationally active, two distinct areas with different translation patterns could be identified after NEBD. In the GV oocyte, the translational activity appeared mainly in the perinuclear area (Fig. 3a). After NEBD, however, we detected two distinct areas with active translation. The first was located in the immediate vicinity of the chromosomes (from here on called chromosomal translational area—CTA), and the second was found in the perispindular area (from here on called perispindular translational area—PTA). (Fig. 3a,b and Supplementary Movie 1). Both the regions were separated by the cytoplasm with a decreased HPG signal. These regions of HPG signal migrated with the spindle to the oocyte cortex and disappeared after cytokinesis (polar body extrusion; MII) (Fig. 3a).

To elucidate the role of these distinctly defined translational regions further, we decided to characterize the localization/distribution of endoplasmic reticulum (ER), which was recently reported to be present at the perispindular area in both oocytes and somatic cells^38^,^39^,^40^. Interestingly, the ER-tracker revealed that the ER formed a circular structure between the CTA and PTA regions with overlaps on the PTA and surrounding cytoplasm (Fig. 3c and Supplementary Fig. 4). Immunostaining of Lamin A/C (LMN) revealed structures surrounding the CTA and present in the gap region with an absence of a nascent HPG translation signal (Fig. 3c and Supplementary Fig. 4). Surprisingly, although the nuclear membrane was already disassembled during NEBD, it appeared that its former structure was subsequently preserved by LMN fragments during pro-metaphase I (pro-MI) before disappearing in the MII stage. The localization of LMN staining between the CTA and PTA regions overlaps with ER-tracker localization (Fig. 3c). Disruption of the microfilament network by cytochalasin D abolished the observed translational pattern as well as LMN from the CTA cortex (Supplementary Fig. 5).

These findings suggest that the oocyte translates *de novo* proteins in distinct locations, which then undergo remodelling at or shortly after NEBD and at cytokinesis (MII). Both ER and LMN are likely to be involved in the formation of the boundary between the two distinct translational areas and probably ensure physical separation of the chromosomes from the rest of the cytoplasm during early stages of meiosis after NEBD. Since the period around NEBD appeared to be crucial both for the translational reorganization and for the timing of spindle assembly, further experiments focused on this stage.

### Components of the mTOR/4F axis are localized to the CTA

Our results thus far led us to hypothesize that the observed phenotype developed due to the defects in the translation of specific mRNAs in specific subcellular compartments. To confirm this hypothesis and to determine whether the mTOR–eIF4F pathway is involved in the CTA and PTA localized translation, we analysed the key components of this pathway at the time of NEBD. Because the mTOR–eIF4F pathway was activated at or shortly after NEBD (when the CTA became apparent), we analysed oocytes 3 h PIW for the presence and localization of eIF4E, phospho-eIF4E, mTOR, phospho-mTOR, phospho-S6K, mTOR’s substrate 4E-BP1 and two differently phosphorylated forms of 4E-BP1.

**Figure 4:**
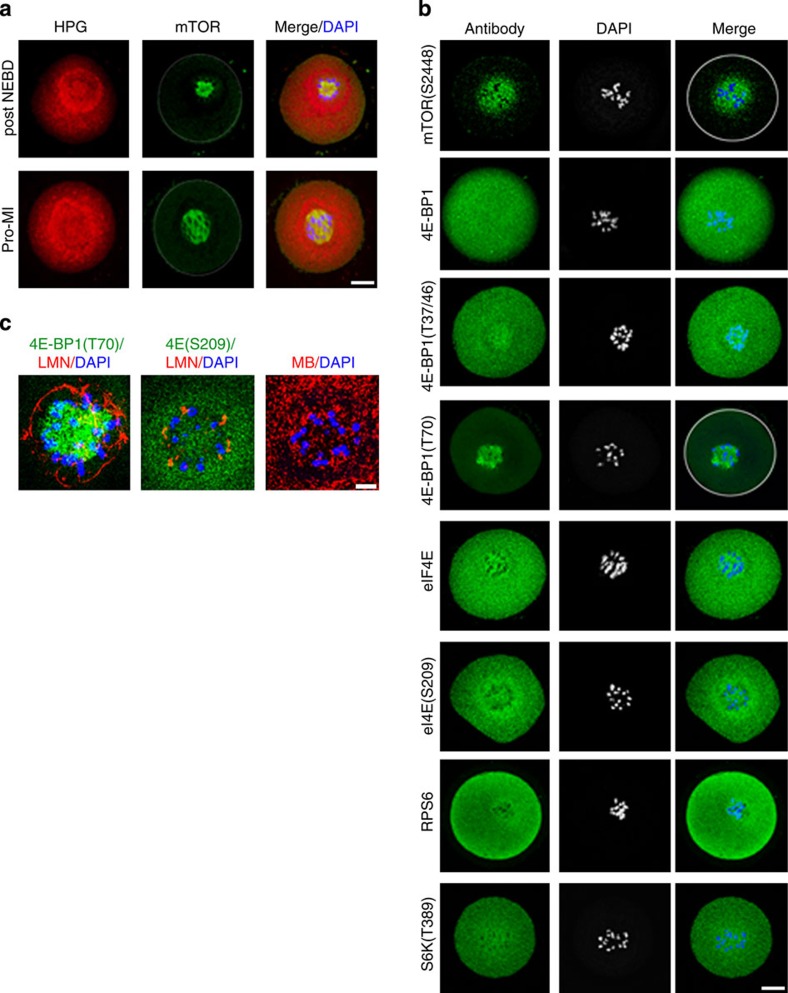
mTOR–eIF4F key players are localized at the CTA. (**a**) mTOR (green) localizes with HPG signal (red) at the CTA. (**b**) Immunocytochemistry shows the localization of mTOR–eIF4F pathway components 2 h post NEBD. White line indicates oocyte cortex; representative images of at least three independent experiments are shown; scale bars, 20 μm. See also Supplementary Fig. 6. (**c**) ER tracker shows perispindular localization of ER and overlaps with the PTA post NEBD. Polymerized LMN separates CTA from PTA. HPG, LMN, MB (red); 4E-BP1(T70), eIF4E(S209) green; ER tracker and DAPI (blue). Data are representatives of at least two independent experiments; white line indicates oocyte cortex; scale bar, ~10 μm.

Both mTOR and mTOR phosphorylated on S2448 (this modification of mTOR was previously linked to the stimulation of translational activity)^41^,^42^ were localized predominantly at the CTA (Fig. 4a,b). However, the analysis of its substrate, 4E-BP1, showed an even distribution within the oocyte. Although its phosphorylated form (T37/46) was localized with a similar pattern asthat of the total protein, significantly higher intensity of the phospho-4E-BP1 signal could be seen in the vicinity of the chromosomes (Fig. 4b). Surprisingly, 4E-BP1 phosphorylated on T70 showed exclusive signal at the CTA (Fig. 4b). Consistent with immunoblot analysis data (Fig. 2a,b and Supplementary Fig. 6), 4E-BP1 was not phosphorylated at the GV stage. The immunofluorescence signal for eIF4E and eIF4E (S209) was localized evenly and it was also present in the vicinity of chromosomes after NEBD. eIF4E phosphorylated on S209 and S6K phosphorylated on T389 also showed an evenly distributed signal in the oocyte. However, in the case of eIF4E (S209), staining could be seen at the CTA and PTA (Fig. 4b,c). Furthermore, the presence of ribosomal protein 6 (RPS6), which has been known to upregulate mRNA translation and can be used as a marker for active translation^43^, was found throughout the cytoplasm as well as at CTA and PTA (Fig. 4b). Additional experiments also show the localization of 4E-BP1(T70) and eIF4E(S209), as well as poly(A)-RNA at the CTA and/or PTA correlating with LMN localization (Fig. 4c and Supplementary Fig. 4). These data clearly demonstrate that the key components of the mTOR–eIF4F pathway are located at the CTA and PTA regions where translation is presumably increased.

### The mTOR–eIF4F pathway regulates translation at CTA

To further elucidate the involvement of the mTOR/4E pathway in the regulation of the translation localized at the CTA and PTA regions, we performed additional experiments utilizing specific inhibitors of this pathway, 4EGI and Rap.

**Figure 5:**
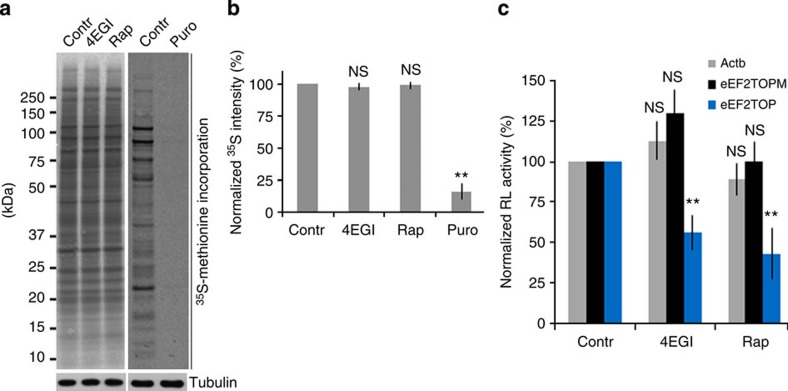
Downregulation of mTOR–eIF4F does not affect global translation, however, shows decreased level of candidate proteins. (**a**,**b**) 4EGI or Rap treatments during meiotic progression do not affect the overall protein synthesis in the oocytes (data are represented as mean±s.d.; **P<0.01, according to Student’s *t*-test; *n*≥3). The overall translation was supressed by puro (puromycin). (**c**) Translation of TOP motive RL RNA reporter construct is affected in the oocytes with downregulated mTOR–eIF4F pathway (as mean±s.d.; **P<0.01; according to Student’s *t*-test.

Incorporation of ^35^S-Methionine in the oocytes treated with 4EGI or Rap during 12 h in meiotic progression revealed no major effect on the overall protein synthesis (Fig. 5a,b). This indicates that the inhibition of the mTOR–eIF4F pathway likely affects translation of only a subset of mRNAs. This was also supported by the previously described experiment in which we analysed the translation of RL RNA reporter constructs microinjected into oocytes. While translation of RL constructs after NEBD containing upstream non-TOP sequence (Actb) or mutated oligopirimidine sequence (eEF2^TOPM^) did not change significantly in oocytes treated with 4EGI or Rap, the translation of the construct containing the canonical oligopirimidine sequence (eEF2^TOP^) was significantly decreased (Fig. 5c).

**Figure 6:**
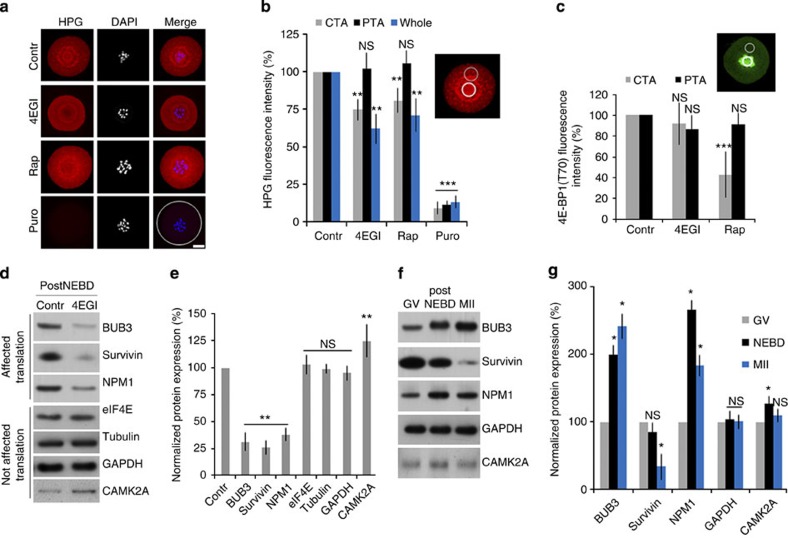
Downregulation of mTOR and 4F abolishes the translation at the CTA. (**a**,**b**) Inhibition of mTOR or 4F decreases HPG fluorescence at the CTA, followed by quantification of HPG fluorescence (bold circle indicates measured area (CTA); thin circle (PTA)). Data are represented as mean±s.d.; **P<0.01 and ***P<0.001, according to a Student’s *t*-test; *n*≥20. Scale bar, 35 μm. The overall translation was supressed by puromycin. (**c**) Measurement of phosphorylation intensity of the 4E-BP1 (T70) shows a decrease at the CTA in the presence of Rap (***P<0.001; *n*≥19). 4EGI does not affect the phosphorylation intensity significantly (*P*>0.1; *n*=20). Bold circle indicates measurement at the CTA and thin circle at PTA/cytoplasm. Data represents the mean ±s.d.; asterisks denote statistically significant differences Student’s *t*-test. (**d**,**e**) Downregulation of the 4F pathway results in decreased translation of selected mRNAs. Data are representative of at least three independent experiments. Values obtained for GV stage were set as 100%. Data are represented as the mean±s.d. **P<0.01, according to a Student’s *t*-test. (**f**,**g**) Immunoblot analysis of the candidate proteins during meiotic maturation. Values obtained for GV stage were set as 100%, *n*≥3. Data represents the mean ±s.d.; asterisk denotes statistically significant differences Student’s *t*-test: *P*<0.05. See also Supplementary Fig. 10.

The timing of NEBD was similar to the control group in both the treatments (Supplementary Fig. 2c). When oocytes were cultured in the presence of HPG and treated with 4EGI or Rap, a significant decrease (~20%; *P*<0.001) in translation fluorescence signal could be seen within the CTA with no visible change in translation within the cytoplasm (Fig. 6a,b). Puromycin is a potent inhibitor of all translations, and treatment on oocytes resulted in the suppression of ^35^S-Methionine incorporation (Fig. 5a,b) as well as signal from HPG (~ 90%; *P*<0.001; Fig. 6a,b).

We further asked whether the downregulation of mTOR–eIF4F would also influence phosphorylation of the mTOR substrate 4E-BP1 on T70 (this modification was detected in our previous experiments to be present exclusively at CTA). Oocytes were cultured in the presence or absence of inhibitors for 3 h PIW and probed for phospho-4E-BP1 (T70). The immunofluorescence signal in the equatorial confocal image section was quantified at the CTA and PTA/cytoplasm. The phospho-4E-BP1 (T70) signal significantly decreased by 57% (*P*<0.001) in the presence of Rap but not 4EGI (Fig. 6c). Interestingly, fluorescence intensity of the PTA/cytoplasm did not change significantly between the groups (Fig. 6a,b). Further support of the effect of Rap brought the immunoblotting experiment showing substantially decreased phosphorylation of 4E-BP1 on T70 in oocytes treated with Rap, but not with 4EGI. On the other hand, 4EGI supressed the formation of the 4F initiation complex (Supplementary Fig. 1a,b) 4EGI. Supression of 4F complex formation did not show an effect on S6K phosphorylation, whereas a mild (30%) effect compared with Hela cells (100%) could be seen when Rap, an inhibitor of mTOR, was used (Supplementary Fig. 1c,d).

Although 4EGI and Rap should not decrease the overall protein synthesis and are supposed to inhibit only cap-dependent translation, we wanted to confirm this. It is known that the eIF4F complex promotes translation of RNAs containing^33^,^44^ TOP. We selected three TOP RNAs, *Bub3, Npm1* (ref. 33) and *Survivin*^45^, whose translation would be negatively affected by the disruption of the eIF4F complex. We analysed protein expression by immunoblotting and found that the translation of selected mRNAs was significantly downregulated (~70% of treated oocytes). On the other hand, translation of TUBA, GAPDH and eIF4E proteins was not influenced by the treatment (Fig. 6d,e and Supplementary Fig. 10). However, the translation of mRNA with an internal ribosome entry site motif for CAMK2A^46^ increased 25%. Translation of BUB3 and NPM1 increased substantially after NEBD, however, the level of Survivin decreased at the MII stage (Fig. 6f,g and Supplementary Fig. 10). Although translation of specific transcripts was decreased in treated oocytes, their mRNA level was not affected (Supplementary Fig. 7) except for *Camk2a*, the mRNA level of which was significantly increased by 4EGI treatment.

These data demonstrate that although the downregulation of mTOR–eIF4F translation in the oocyte does not influence the overall translational pattern, protein synthesis at the CTA region is impaired.

### The oocyte nucleus stores a large pool of RNA

**Figure 7:**
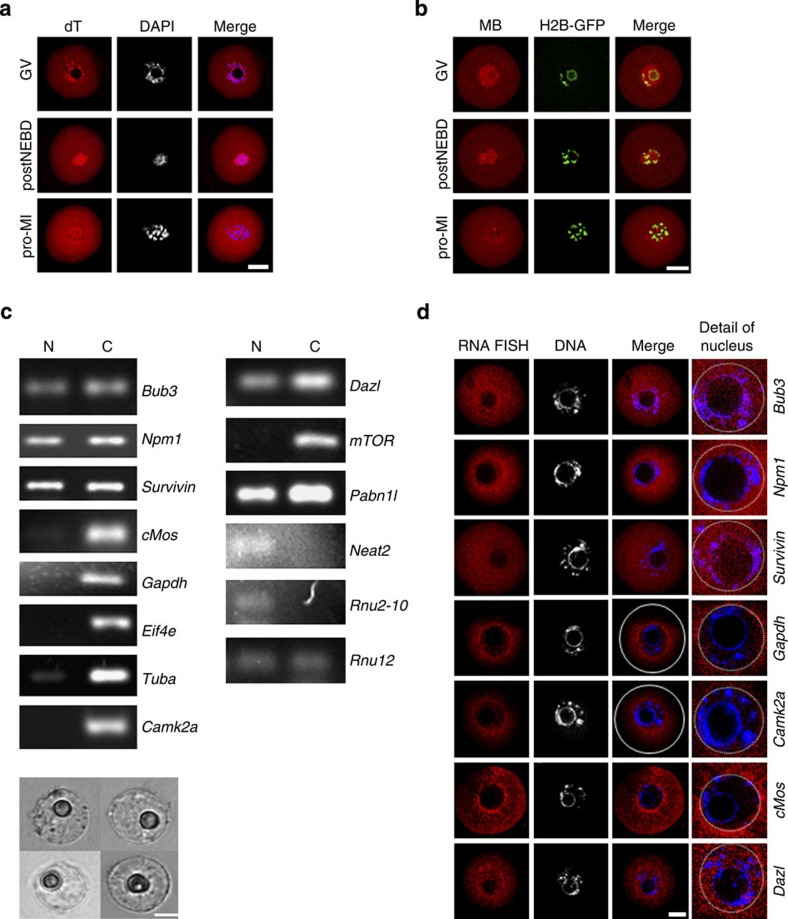
The oocyte nucleus stores a pool of RNA. (**a**) RNA FISH shows the presence of a poly(A)-RNA population in the nucleus and in the vicinity of chromosomes in GV and NEBD stage oocytes. Poly(A) (red); DAPI (Blue). See also Supplementary Fig. 8. Scale bar, 20 μm. (**b**) MB shows the presence of poly(A)-RNA in the nucleus and in the vicinity of chromosomes in the GV oocyte and 2 h after NEBD. MB (red); H2B-GFP (green fluorescent protein; green). Scale bar, 20 μm. See also Supplementary Movie 2. (**c**) The oocyte nucleus contains specific mRNAs. RNA isolated from the nuclear (N) and cytosolic (C) fractions shows the presence or absence of specific transcripts by PCR. Data represents at least three independent experiments. Representative images of the isolated nuclei from mouse oocytes are shown. Scale bar, 10 μm. See also Supplementary Fig. 9 and Supplementary Table 1. (**d**) RNA FISH shows nuclear absence of *Camk2a, cMos, Gapdh* and nuclear presence of *Bub3, Nph1, Survivin, Dazl* mRNAs. The white line indicates the border of oocyte cortex; the white dashed line indicates the border of the nucleus; representative images of two independent experiments shown. Bar size, 20 μm.

We further determined the role of RNA localization in the translation detected at the CTA and PTA regions. Since it has been shown that mRNA localization generally leads to targeted translation^47^,^48^,^49^, we labelled the poly(A)-RNA population with an oligo dT probe to detect mRNA localization in the oocyte via fluorescence *in situ* hybridization (FISH). Surprisingly, we detected a strong signal of endogenous poly(A)-RNAs in the nucleus of the fully grown oocyte (Fig. 7a). RNase treatment resulted in a decrease in the FISH signal, while treatment with DNase did not abolish the poly(A)-RNA signal in the oocyte (Supplementary Fig. 8a). After NEBD, a strong signal corresponding to poly(A)-RNA could be detected in the vicinity of chromosomes matching precisely to the CTA region and to a lesser extent in the cytoplasm. In the pro-MI stage, the poly(A) signal was still present at the CTA (Fig. 7a). The poly(A)-RNA population in the nucleus of growing oocytes appeared diffuse in comparison to fully grown oocytes (Supplementary Fig. 8b).

To confirm the data from RNA FISH in live oocytes we performed experiments using a molecular beacon probe^50^ (MB), which under *in vivo* conditions was able to hybridize to the poly(A) stretch of endogenous RNA. Oocytes in the GV stage were microinjected with a MB probe and the distribution of poly(A)-RNA was followed by live-cell imaging. The results obtained with the MB probe injected into live oocytes were consistent with RNA FISH results (Fig. 7b and Supplementary Movie 2). Furthermore, staining of the oocytes with the nucleic acid marker SYTO14 also showed the presence of fluorescence signals in the nucleus of oocytes and also in the vicinity of chromosomes during MI in a region consistent with the CTA (Supplementary Fig. 8c).

Finally, we also investigated, whether the nucleus of a fully grown oocyte contained specific mRNAs, especially those coding for proteins affected by the 4EGI inhibitor (Fig. 6d,e). We isolated RNA from the oocyte nuclei (Fig. 7c) and cytoplasms and performed PCR for selected RNAs known to be present in the nucleus^51^. Our data clearly showed the presence of *Bub3, Npm1, Survivin, Dazl* and *Pabn1l* mRNAs in both the nucleus and cytoplasm (Fig. 7c), while other transcripts, such as *Mos, Gapdh, Tuba, mTOR, Eif4e* and *Camk2a*, were present only in the cytoplasm and were excluded from the nucleus. We also looked for the presence of known transcripts localized to the nucleus such as non-coding RNAs (*Neat2, U2* and *U12)* and *Pabpnl1* mRNA^52^,^53^ (Fig. 7c). The presence or absence of mRNAs in the oocyte nucleus was also visualized by single-molecule RNA FISH (Fig. 7d), and the results showed that while *Camk2a*, *Mos* and *Gapdh* mRNA were absent, *Bub3, Npm1, Survivin* and *Dazl* mRNAs were localized to the nucleus.

Taken together, our data indicate that the oocyte nucleus contains an RNA population that most likely contributes to translation in the vicinty of chromosomes after NEBD.

## Discussion

Post-transcriptional control of gene expression at the level of translation has been shown to be essential for regulating a number of cellular processes during development^1^. This is especially true in mammalian oocytes which, after a transcriptionally active period during their growth, resume meiosis during a period of transcriptional quiescence with a store of maternally synthesized mRNAs. Progression through meiosis is therefore regulated in the oocyte at the level of mRNA stabilization, translation and post-translational modification.

The importance of protein synthesis for meiotic and mitotic progression has been shown previously. Those published results revealed that protein synthesis is not required for NEBD in mouse oocytes, although the formation of the spindle and progression to metaphase II requires active protein synthesis^54^. This requirement for global translation has been attributed mainly to the activation of maturation-promoting factor, the key regulator of M-phase entry. However, the identity of the proteins, which need to be synthesized, and the spatiotemporal regulation of translation in the oocytes, is not entirely clear.

In this study we show that the disruption of mTOR–eIF4F signalling (playing a central role in the regulation of cap-dependent translation)^28^,^33^,^55^ does not impair the oocyte meiotic progression to metaphase II. However, it leads to severe defects in the spindle morphology and chromosome alignment in metaphase I and II resulting in chromosomal aneuploidy. This suggests that activation of the mTOR–eIF4F signalling pathway is not required for maturation-promoting factor activation, but it is important for the synthesis of specific proteins that are required for the normal function of the spindle and proper distribution of the chromosomes during meiosis I. Disruption of the mTOR–eIF4F signalling pathway does not visibly influence the overall translation. Instead, we observe the downregulation of translation of a subset of specific mRNAs. This indicates that translation of the vast majority of mRNAs is regulated through other mechanisms^56^. It has been shown that translation of *Bub3, Npm1* and *Survivin* mRNAs is regulated by the 4F complex^33^,^45^. BUB3, NPM1 and Survivin play roles in spindle assembly and chromosome alignment and thus in the maintenance of genomic stability^57^,^58^,^59^,^60^. Both BUB3 and NPM1 are increasingly translated after NEBD; however, the translation of Survivin decreases in MII suggesting rapid protein turnover in the oocyte^61^. *Camk2a* mRNA with an internal ribosome entry site motif^45^ revealed that its translation is not affected after inhibition of 4F formation, which positively correlates with the results obtained using a RL reporter of mRNAs without TOP or with mutated TOP motifs. On the other hand, *Camk2a* shows higher stability after 4EGI treatment suggesting that the active translation exerts a protective effect on mRNA from decay. Despite aberrant translation of selected transcripts, meiotic progression is unaffected probably due to the altered spindle assembly checkpoint regulatory mechanism in oocytes^62^. Consistent with this, defects in spindle morphology and chromosome alignment have been observed.

We show that activation of the key components of the mTOR–eIF4F pathway and translation of RNAs with a 5′ TOP motif after NEBD in oocytes and inactivation after fertilization (entry to interphase) that indicates a role in cell cycle progression. Furthermore, we have detected nascent translation with surprisingly precise localization of two particular ‘translational hotspots’. These newly described areas with an increased level of translation, one in the vicinity of chromosomes and another around the spindle (perispindular area), have been designated as the CTA and PTA, respectively. We have further shown that both mTOR and phosphorylated (active) mTOR, as well as eIF4E and phospho-eIF4E, are predominantly localized to the CTA. Similar localization has also been observed for the mTOR direct target, 4E-BP1, with protein phosphorylation on T37/46. T70 phospho-4E-BP1 is present almost exclusively at the CTA and this phosphorylation is affected by the mTOR inhibitor Rap. Consistent with these results, the distribution of differently phosphorylated forms of 4E-BP1 and RPS6 during mouse oocyte meiotic progression has been recently described^27^. The phosphorylation of RPS6 contributes to the formation of translation initiation complexes and the formation of polysomes^63^,^64^, and it correlates with an increase in translation of 5′TOP mRNA sequences^65^,^66^, thus it is commonly used as a marker of active translation. Another branch of the mTOR pathway is S6K; however, S6K in our model system is already highly phosphorylated at the GV stage and then its phosphorylation significantly decreases during meiotic maturation. Our data positively correlate with data published previously^67^ showing that during cell cycle progression the inactivation of S6K presumably serves to spare energy for costly cell cycle processes at the expense of ribosomal protein synthesis. Moreover, the gradual decrease in the S6K activity during oocyte maturation can also explain our previously published data^26^, showing that the overall protein synthesis decreases during meiotic maturation of porcine oocytes while both eIF4E and 4E-BP1 become phosphorylated during this period. Upon treatment with Rap we observed only a minor (30%) decrease in S6K phosphorylation when compared with the control oocytes. A possible explanation for this rather unusual observation might be sequence divergence of the region encompassing the Ser/Thr phosphorylation site of S6K in oocytes compared with somatic cells, which could cause partial insensitivity to Rap treatment^68^.

Our data, along with those published by others, indicate that the key components of the mTOR–eIF4F pathway (as markers of active cap-dependent translation) play an important role at the CTA, and that this localization is essential for translation of specific RNAs involved in the correct formation of the spindle and accurate positioning of chromosomes. This idea is also supported by our data showing that the inhibition of the mTOR–eIF4F pathway (either by 4EGI or Rap treatment) leads to an abolition of translation at the CTA.

The region between the CTA and PTA with diminished translation contains ER and LMNs. We hypothesize that this gap between the translational active areas is some sort of semipermeable membrane formed on the basis of microfilaments^69^,^70^, ER^38^,^39^,^40^, LMN and possibly other constituents. This structure becomes apparent in the fully grown GV oocyte^38^,^70^ with the PTA. We also hypothesize that this structure plays a role after NEBD onset to prevent rapid escape of nuclear components (mRNAs, ncRNAs, nuclear proteins and chromosomes) to the cytoplasm of such a large cell (~70 μm) and/or to prevent the entry of the cytoplasmic elements into the CTA, successfully maintaining organelle compartmentalization. Spatial translational control may provide an important means to maintain and refine these patterns of expression over time. Indeed, the distribution of certain transcripts and proteins appears to be distinct. This may contribute to spindle and chromosome organization and play an important role in the maintenance of genomic stability.

Previously, it has been reported that an abundant RNA population with RNA-binding proteins is localized to the cortical region of the oocyte^13^,^14^. This would, however, suppose that the RNAs or their products have to undergo massive changes in localization to ensure non-erroneous regulation of all the morphological changes occurring during meiotic progression. Alternatively, our results reveal a markedly enriched population of poly(A)-RNAs present in the nucleus of the fully grown oocyte without significant subcortical enrichment. In addition, mTOR–eIF4F axis components are not enriched in the subcortical region. Using multiple independent methods, we document the presence of endogenous RNAs in the nucleus of the oocyte that persist after NEBD in the vicinity of the condensed chromosomes overlapping with the CTA region. We believe that the observed nuclear localization of RNAs is a mechanism to ensure temporal and spatial translation of mRNAs important for the onset and progression of the dynamic processes of meiosis, especially spindle assembly. The oocyte nucleus seems to serve as a reservoir of transcripts retained during the transcriptionally active phase, and this finding positively correlates with protein localization at the spindle/chromosome area during cell cycle^59^,^71^,^72^. This hypothesis is supported by our results showing the presence of selected transcripts in the nuclei of a fully grown oocyte. Importantly, it has been shown that RNA is not translated following injection into the nucleus, but it is translated after NEBD^73^. Oocytes before NEBD are unsuitable as recipients for nuclear transfer, leading to abnormal cell division^74^,^75^. Our research demonstrates that this could be caused by the fact that the nucleoplasm contains a rich RNA population that resembles a ‘nuclear factor’ essential to support oocyte maturation and early embryo development. The oocyte’s nuclear transcriptome remains to be described. These results suggest that the function of mRNA retention in the nucleus may be to sustain translational repression, and that their subsequent translation can be regulated in a spatiotemporal restricted manner in response to cell cycle events.

Preserving the localization of specific translational factors and RNAs in specific cell compartments (chromosomes and newly forming spindle) at the onset of meiosis contributes to a less error-prone cell cycle progression in such a large cell. Moreover, the preservation of LMN and ER structures after NEBD posibly contributes to cytoplasm fractionation and ensures organelle compartmentalization. It is well-known that the nucleus contains various RNA species (coding and non-coding) that might also contribute to localized translation after NEBD^51^,^52^,^53^. Understanding the mechanisms whereby mRNAs are localized and their translation is locally regulated thus promises to provide important insights into many aspects of cell physiology.

Major causes of human aneuploidy involve errors that arise during meiosis^76^. Our data suggest that misslocalization of specific transcripts within the oocyte and their aberrant translation could be another cause of aneuploidy. This work describes components that are potential clinically relevant targets.

Altogether, our findings indicate that a nuclear RNA population contributes to mammalian oocyte translational patterning and thus to the regulation of gene expression during the dynamic onset of meiosis. At the molecular level, we present an important function for the mTOR–eIF4F pathway in spatial translational control, suggesting a novel set of regulatory mechanisms ensuring specific gene expression at the right place and time in the mammalian oocyte.

## Methods

### Oocyte culture and microinjection

GV oocytes were obtained from at least 6 week-old CD1 mice 46 h after injection of pregnant mare serum gonadotropin (PMSG). Oocytes were placed in M2 medium (Millipore) supplemented with 100 μM of IBMX ((3-isobutyl-1-methylxanthine, phosphodiesterase inhibitor; Sigma)) to prevent NEBD. Selected oocytes were denuded and cultured in M16 medium (Millipore) without IBMX at 37 °C, 5% CO_2_. After IBMX wash (PIW) at least 90% of oocytes resume meiosis (NEBD) within 70 min. To obtain MII oocytes, hCG (Sigma) was administered 48 h after PMSG. Zygotes were obtained from the PMSG-primed females mated to males 17 h post hCG. Oocytes were microinjected by Narishige microinjector with ~5 pl of the solution containing 20–50 ng μl^−1^ RNA per oocyte and cultured according to the protocol. Oocytes were treated with of 100 μM 4EGI (Calbiochem), 100 nM Rap, 3 μg ml^−1^ CCD or 1 μg ml^−1^ puromycin (Sigma). Dimethylsulphoxide was used as a control. All animal work was conducted according to Act No 246/1992 on the protection of animals against cruelty. Hela cells were cultured in DMEM F12 with 5% fetal bovine serum, 1%penicilin/streptomycin, 1% Glutamax and with presence or absence of 100 nM Rap for 3 h.

### Immunocytochemistry and fluorescent probe detection

Oocytes were fixed in 4% paraformaldehyde (PFA) in PBS for 30 min, permeabilized for 15 min in PBS with 0.1% Triton X-100 and incubated overnight at 4 °C with primary antibodies (1:100) against 4E-BP1(T70), 4E-BP1(T37/46), S6K(T389; Cell Signaling Technology), RPS6 (Santa Cruz), LMN A/C or α-tubulin (Sigma). After washing, the oocytes were incubated for 1 h at room temperature with an Alexa Fluor conjugated antibodies (1:250; Molecular Probes). RNaseOut (500 U ml^−1^; Invitrogen) was used in all the buffers. For nascent protein synthesis specific stage (GV-0 h, NEBD-2 h, pro-MI-7 h, MII-12 h) oocytes were cultured in the methionine-free medium (Gibco) supplemented with 1% dialyzed fetal bovine serum (10,000 MW; Sigma) and 50 μM HPG for 30 min^77^. HPG was detected by using Click-iT Cell Reaction Kit (Life Technologies). Chromosome spreads from mouse oocytes were prepared as previously described^78^. ER was detected by 1 μM ER-Tracker (Green dye and Blue-White DPX dye for double staining; Molecular Probes) in M16 for 1 h. DAPI was used for chromosome staining (Vectashield). Nucleic acids were labelled by 50 nM SYTO14 (Molecular Probes) in M16 for 20 min then fixed by PFA and imaged. Samples were visualized using an inverted confocal microscope in 16 bit depth (TCS SP5; Leica). Images were assembled in Photoshop CS3 and quantified by Image J software.

### Measurement of overall protein synthesis

To measure the overall protein synthesis, 50 μCi of ^35^S-methionine^79^ (Perkin Elmer) was added to methionine-free culture medium. Twenty-five oocytes per sample were labelled for 12 h, then lysed in SDS-buffer and subjected to SDS–polyacrylamide gel electrophoresis (PAGE). The labelled proteins were visualized by autoradiography on BasReader (Fuji) and quantified by Aida software (RayTest). Tubulin was used as a loading control.

### Immunoblotting

Oocytes were lysed in 10 μl of 1 × Reducing SDS Loading Buffer (Cell Signaling Technology) and heated at 100 °C for 5 min. Proteins were separated by gradient 4–20% SDS–PAGE and transferred to Immobilon P membrane (Millipore) using a semidry blotting system (Biometra GmbH) for 25 min at 5 mA cm^−2^. Membranes were blocked, depending on the used antibody, in 2.5 or 5% skimmed milk dissolved in 0,05% Tween-Tris-buffer saline (TTBS), pH 7.4 for 1 h. After a brief washing in TTBS, membranes were incubated at 4 °C overnight with the following primary antibodies with 1% milk/TTBS: mTOR(1:8,000), mTOR-S2448 (1:8,000), eIF4G1-S1108 (1:1,000), eIF4E-S209 (1:1,000), 4E-BP1 (1:500), 4E-BP1-T70 (1:500), 4E-BP1-T36/47 (1:500), eIF4G2 (1:500), eIF2a (1:500), eIF2a-S51 (1:500), S6K (1:2,000), S6K-T389 (1:500), Survivin (1:2,000), CAMK2A (1:1,000) from Cell Signaling Technology; eIF4G1 (1:500), eIF4E (1:500), BUB3 (1:500), from BD; NPM1 (1:500) from Life Technologies, α-Tubulin (1:7,500) from Abcam and GAPDH (1:30,000) from Sigma. Immunodetected proteins were visualized by ECL kit (Amersham), films were scanned using a GS-800 calibrated densitometer (Bio-Rad and quantified using Image J (http://rsbweb.nih.gov/ij) software.

### Live-cell imaging

Oocytes 1–2 h after microinjection were transferred in M16 medium to Leica SP5 confocal microscope equipped with EMBL stage incubator and HCX PL APO 20 × /0.7 IMM CORR λ_BL_ and HCX PL APO 40 × /1.1 Water corrected objectives. MB (2′OME-RNA: Cal Fluor Red 635-GCACGT-(U)_20_–ACGTGC–3′BHQ2) probe (Biosearch Technologies) was injected in 20 μg μl^−1^ concentration with non-polyadenylated H2B:GFP RNA^80^. Movie was assembled using Image J.

### Polymerase chain reaction

RNA was extracted with RNeasy Plus Micro kit (Qiagen). Genomic DNA was depleted using columns (Supplementary Fig. 9). Primers were designed in two exons flanking introns (Supplementary Table 1). Reverse transcription with Sensiscript RT kit (Qiagen). PCR program used: 95 °C/30 s, (95 °C/30 s, 60 °C/90 s for *Rnu2-10* and *Mos*, for other genes 58 °C/90 s, 72 °C/90 s) × 35 cycles, 72 °C/5 min. Products were detected by electrophoresis on 1.2% agarose gel with ethidium bromide. RT–PCR was carried out on Rotor-Gene 3000 (Corbett Research) using OneStep RT–PCR Kit (Qiagene) and SybrGreen, data was analysed by internal software Rotor-Gene 3000. Reaction conditions were: RT 50 °C/30 min, initial activation 95 °C/15 min, (95 °C/15 s, annealing at a temperature specific for each set of primers (see Supplementary Table 1)/20 s, 72 °C/30 s) × 40 cycles, 72 °C/10 min.

### Dual-luciferase assay

Oocytes were injected with 50 ng μl^−1^ *in vitro* trascribed RNA (T7 mMessage, Ambion) from Renilla Luciferase constructs (RL; # 38234, 38235, 38236, Addgene^81^; pRL-EMCV^82^) with combination of injection amount control Firefly Luciferase (FL; # 18964; Addgene) in the presence of IBMX. Oocytes were cultured for 5 h with or without IBMX. At least five oocytes were lysed in 5 μl of Passive Lysis Buffer and stored at −80 °C until luciferase activity was measured by the Dual-Luciferase Assay System (Promega) according to the manufacturer's instructions. Signal intensities were measured using a Glomax Luminometer (Promega). Activity of RL was normalized to that of FL luciferase.

### Chromosome spreads

Zona pellucida was removed by Tyrode acid solution (polar bodies had become detached), washed with M2 medium and subsequently placed into hypotonic solution (1% fetal calf serum in deionized H_2_O). Hypotonic treatment was carried out for <1 min at room temperature. For fixation, oocytes were transferred into 50 μl drop of solution (0.1% paraformaldehyde, 0.2% Triton X-100, 1 mM dithiothreitol, adjusted to pH 9.2 with NaOH) in glass slide (Fisher Scientific). Fixation was allowed to proceed overnight at room temperature. Slides were dried and mounted in Vectashield with 4',6-diamidino-2-phenylindole (DAPI), covered with a glass coverslip and kept at 4 °C. Samples were visualized using an inverted confocal microscope (TCS SP5; Leica) with × 63 objective.

### RNA FISH

RNA FISH was performed according to ref. 83, briefly: 4% PFA fixed and permeabilized by 0.1% Triton X-100 in PBS with RnaseOut (Life Technologies), oocytes were washed with the washing buffer (15% formamide-Sigma, 2xSSC in RNAse free water) and hybridized with 100 nM probe in hybridization buffer (15% formamide-Sigma, 0.1% dextran sulfate, 1 mg ml^−1^ *E.coli* tRNA-Roche, 2 mM Vanadyl-ribonucleoside complex-NEB, 2xSSC in RNAse free water), dT_(22)_, *Bub3, Nph1, Survivin, Gapdh, Camk2a, cMos* and *Dazl* (Biosearch Technologies) labelled with Cy5 or Cal Fluor Red 635 fluorophores for single-molecule RNA FISH at 30 °C overnight. After three washes, the oocytes were mounted into a medium with DAPI (Vectashield). RNase A or DNase (25 μg ml^−1^ for 30 min at 37 °C; Qiagen) was used after the permeabilization step in controls.

### Nuclei isolation

Zona pellucida was removed using Tyrode acid solution (Sigma). The oocytes were disrupted in 100 μl Nuclei EZ lysis buffer (Sigma) and washed four times by centrifugation (2,000 *g* for 4 min at 4 °C). Nuclei sediment and cytoplasm fraction was collected and frozen.

### Immunoprecipitation

Oocytes were lysed in lysis buffer containing 0.5% Triton X-100, 5 mM Tris, 1% deoxycholate sodium salt, 0.15 M NaCl, 1 mM Na_3_VO_4_, 4 mM protease inhibitors (Roche), pH 7.5. After centrifugation at 10,000 *g* for 10 min at 4 °C, the supernatants from 300 post-NEBD oocytes were incubated with 20 μl washed protein agarose beads (Sigma Aldrich) and agarose conjugated with eIF4E antibody (P-2, Santa Cruz) for 12 h at 4 °C. After centrifugation, the bead pellets were washed with ice cold lysis buffer for 5 min three times. Oocyte extracts incubated with resin without antibody was used as a negative control. The SDS-denatured agarose beads were separated by SDS–PAGE and analysed by immunoblotting.

### Statistical analysis

Mean and s.d. values were calculated using MS Excel, statistical significance of the differences between the groups were tested using Student’s *t*-test and *P*<0.05 was considered as statistically significant.

## Author contributions

A.S. and M.K. designed the experiments, carried out the data analysis and planed the project. A.S., D.J., R.C., T.T. and J.S. caried out most of the experiments; A.D. and M.A. caried out live-cell immaging; M.S.C. and J.S.O. performed experiments that contributes intellectually but did not result in figures; R.M. caried out Dual-luciferase assays; A.S. and M.K. wrote the manuscript.

## Additional information

**How to cite this article:** Susor, A. *et al*. Temporal and spatial regulation of translation in the mammalian oocyte via the mTOR–eIF4F pathway. *Nat. Commun.* 6:6078 doi: 10.1038/ncomms7078 (2015).

## Supplementary Material

Supplementary Information Supplementary Figures 1-10 and Supplementary Tables 1

Supplementary Movie 1 Projection of HPG signal in the oocyte after NEBD. HPG (red); DAPI (blue). Z = 40 μm; step 2 μm; scale bar 20 μm

Supplementary Movie 2 Nucleus of a fully grown oocyte contains an abundant RNA population. Time lapse confocal microscopy of a live oocyte imaging Poly(A)-RNA at the resumption of meiosis. Nucleus projection (Z = 10 μm); time point 1 min for 3 h; H2B:GFP (green); MB (red); bar size 20 μm.
