# Supplementary material for: Temporal and spatial regulation of translation in the mammalian oocyte via the mTOR–eIF4F pathway
Source: Nat Commun. 2015 Jan 28;6:6078. doi: 10.1038/ncomms7078 (PMC4317492; doi:10.1038/ncomms7078)
Supplement: Supplementary Information — Supplementary Figures 1-10 and Supplementary Tables 1 [file ncomms7078-s1.pdf]

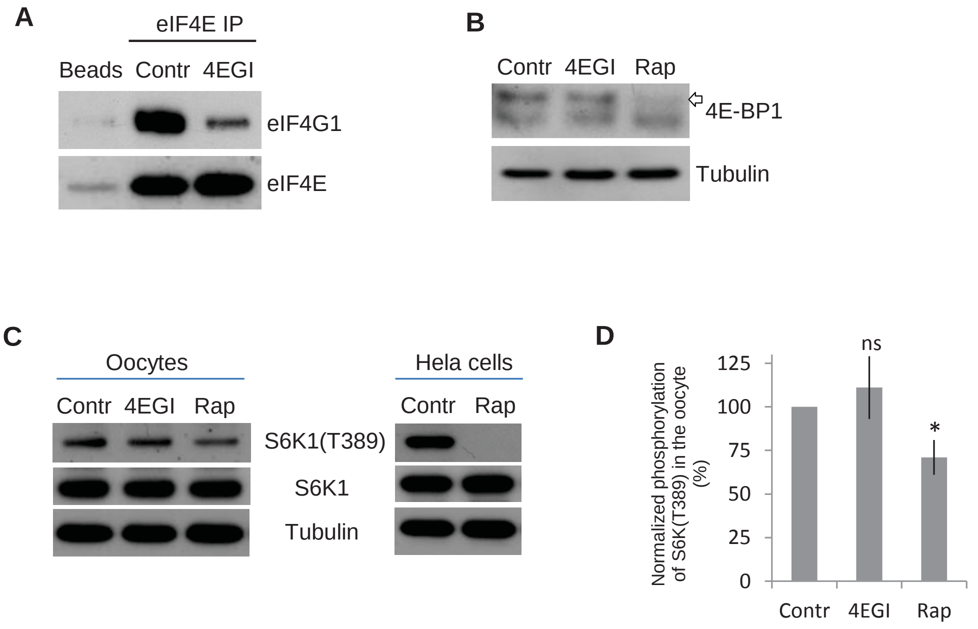

Supplementary Figure 1
4EGI and Rap effectively suppress the mTOR/4F pathway. (**A**) 4EGI suppresses formation of the 4F complex in treated oocytes (n ≥ 2). Oocyte extracts incubated with resin without antibody were used as a negative control. (**B**) IB analysis shows decreased phosphorylation of 4E-BP1 in Rap treated oocytes (n ≥ 2). (**C,D**) IB analysis shows a mild effect of Rap on S6K phosphorylation in oocytes compared to Hela cells. Data are represented as the mean ± SD. Asterisk denotes $p<0.05$; ns = non significant; according to a student's t-test; n = 3.

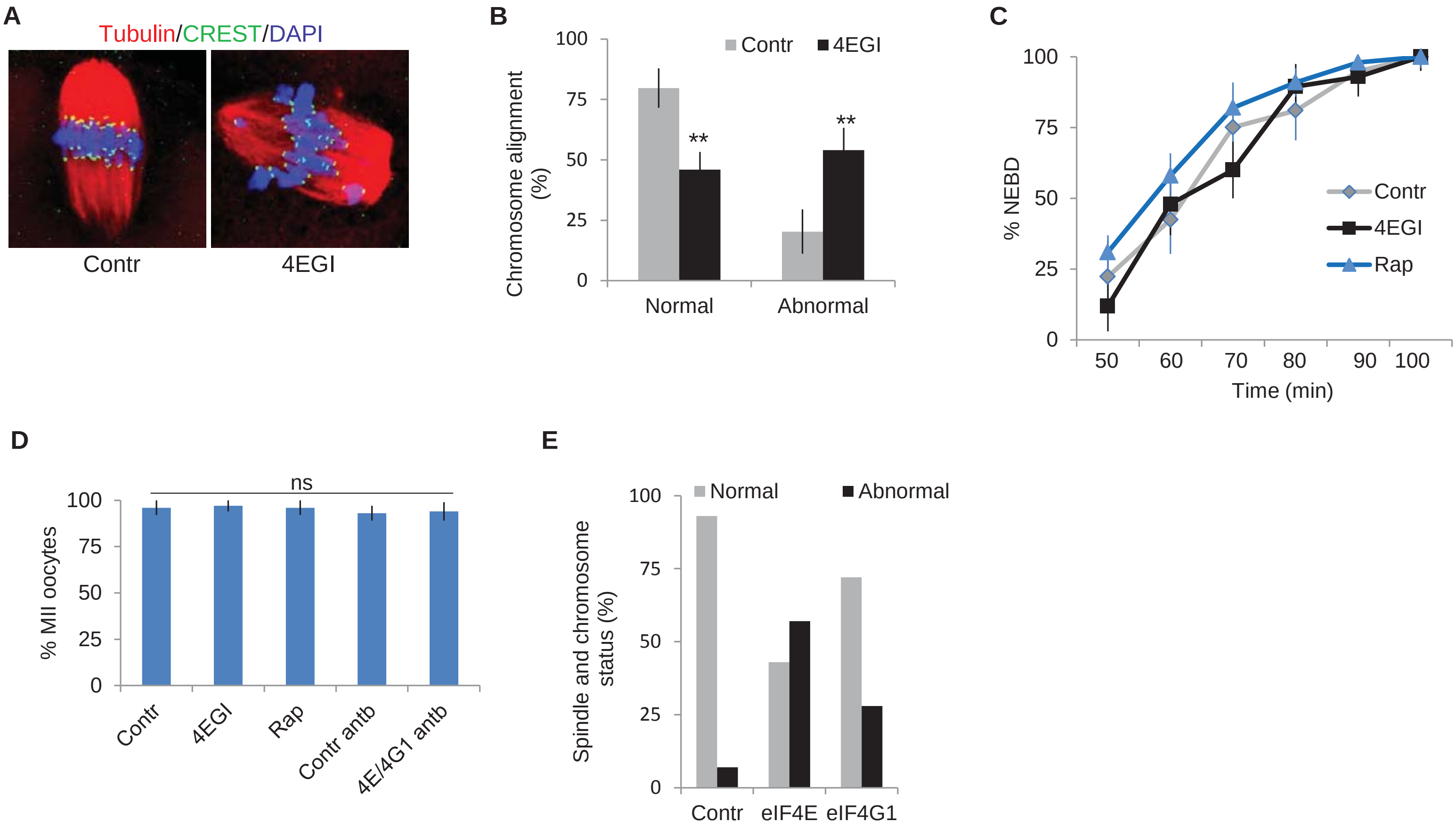

Supplementary Figure 2

Downregulation of the mTOR/4F pathway does not abolish NEBD or cytokinesis (PBE) in mouse oocytes. (**A,B**) Alignment of chromosomes in metaphase I is dramatically affected by 4EGI. Data represents the mean ± SD; asterisks denote statistically significant differences (Student's t-test: $p<0.01$); data represent three independent experiments; n≥22; tubulin (red), centromeres/CREST (green; Immunovision) and DAPI (blue). (**C**) Timing of NEBD in oocytes is not affected by 4EGI or Rap. Data are shown as the mean ± SEM of two independent experiments. (**D**) Rate of polar body extrusion/cytokinesis is not affected in oocytes with down-regulated mTOR/4F. Data represents the mean ± SD; ns denotes non significant, $p>0.05$; Student's t-test; n≥3 independent experiments. (**E**) Microinjection of oocytes with a single antibody against eIF4E or eIF4G1 results in abnormalities of the spindle and chromosome alignment in MII oocytes. From two independent experiments, n≥20.

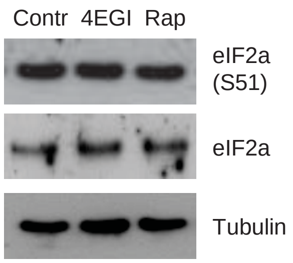

Supplementary Figure 3
4EGI and Rap do not induce an increase in phosphorylation of eIF2a (Ser51). Representative IB is shown from two independent experiments.

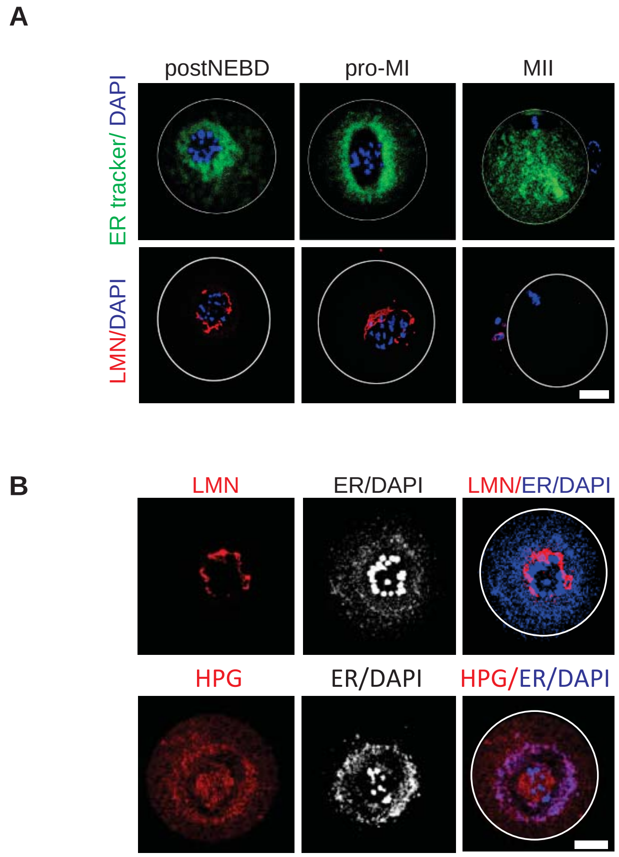

Supplementary Figure 4

ER tracker shows perispindular localization of ER in the oocyte. (**A**) Localization of ER tracker (green) and LMN (red) during meiotic maturation; DAPI (blue). Polymerized LMN is present around chromosomes and disappears after cytokinesis. (**B**) Double labeling of the oocytes postNEBD shows separation of two translational hotspots by LMN and ER surrounding CTA and colocalizing with PTA. HPG and LMN (red); ER tracker and DAPI (blue). Data are representative of at least two independent experiments; white line indicates oocyte cortex; scale bar approximately 20 μm.

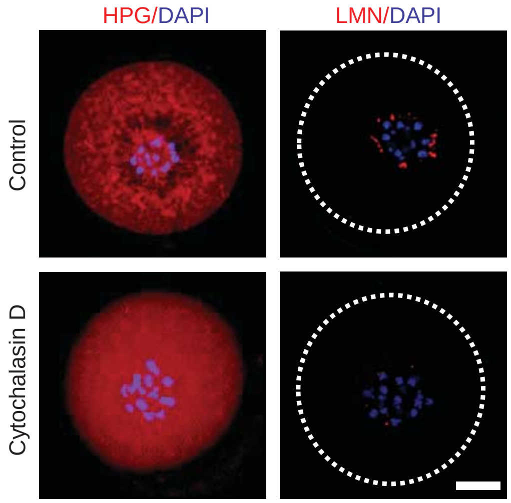

Supplementary Figure 5
Disruption of actin affects the translational pattern and retention of LMN in the vicinity of chromosomes. Oocyte treatment with 3 μg/ml cytochalasin D results in disruption of CTA and PTA and leads to a massive decrease of immunofluorescence signal of LMN in the vicinity of chromosomes. The white line indicates the oocyte cortex; representative images of two independent experiments are shown. HPG and LMN (red); DAPI (blue). Scale bar 20 μm.

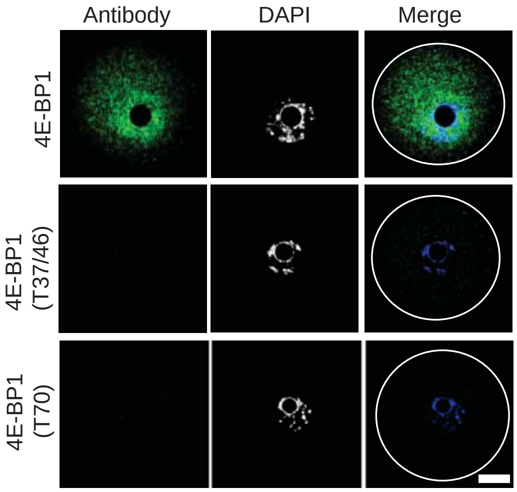

Supplementary Figure 6

4E-BP1 is unphosphorylated on Thr 37/46 and Thr70 at the GV stage. ICC analysis of GV oocytes shows absence of immunofluorescence signal for 4E-BP1(T37/46) and 4E-BP1(T70). Scale bar 20 μm.

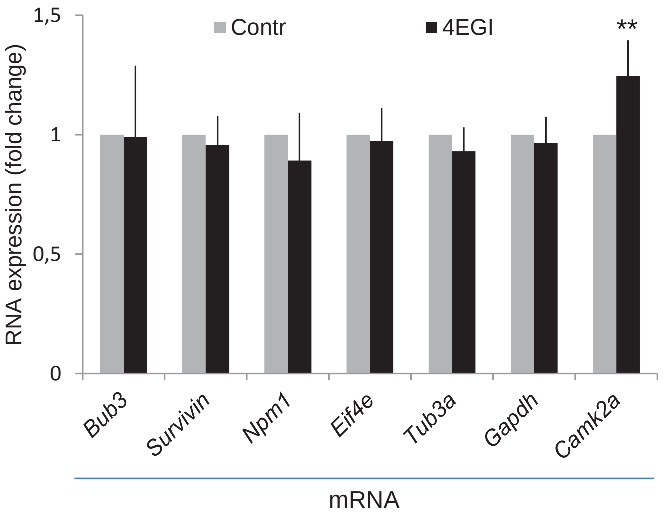

Supplementary Figure 7

Despite the decrease in protein levels of BUB3, NPM1 and Survivin, their mRNA levels remain unchanged during meiotic progression. RT-PCR analysis of *Bub3, Survivin, Npm1, Eif4e, Tub3a* and *Gapdh* shows no significant changes at the mRNA level, only a higher level of *Camk2a* is present after 4EGI treatment. Data represent the mean ± SD; asterisks denote $p<0.01$; bars without asterisks are not significant, Student's t-test; $n \geq 3$ independent experiments.

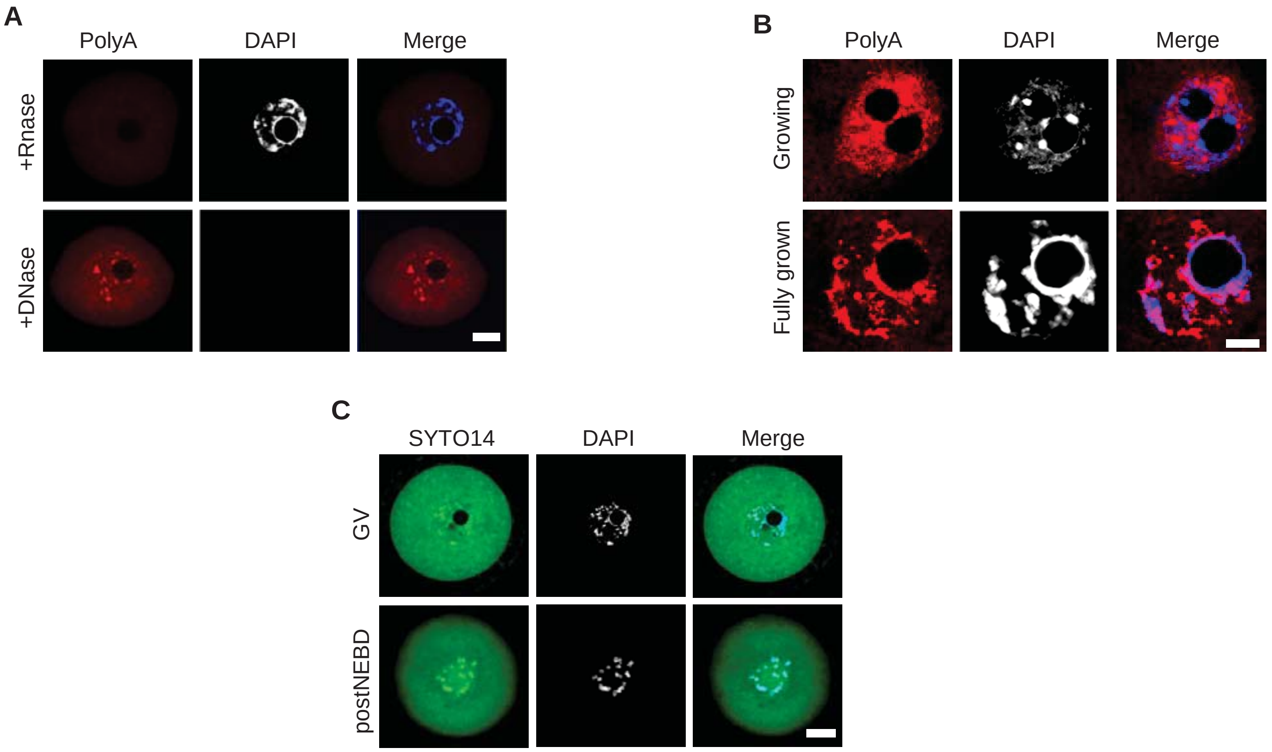

Supplementary Figure 8
The poly(A)-RNA signal differs between growing and fully grown oocytes. (**A**) RNA FISH shows differences in transcriptionally active growing oocytes compared to mature GV oocytes. Scale bar 5 µm. (**B**) Digestion of RNA in the oocyte abolishes poly(A)-RNA fluorescence signal, whereas digestion of DNA does not impair the RNA FISH signal in the oocyte. Scale bar 10 µm. Representative images of two independent experiments are shown. (**C**) Staining of nucleic acids by SYTO14 in the oocyte shows a similar RNA pattern as RNA FISH or MB. Representative images of two independent experiments are shown. Scale bar 20 µm.

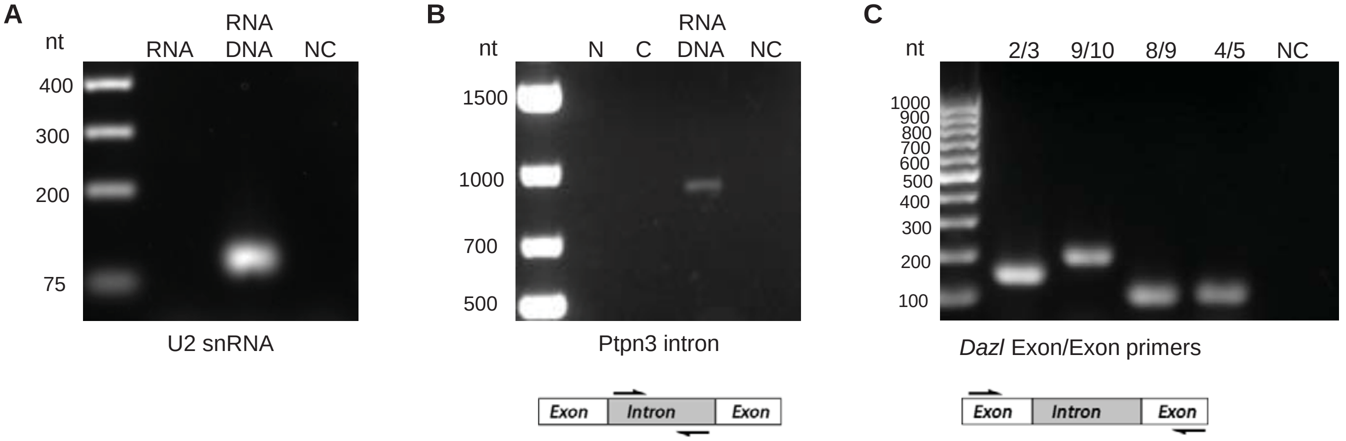

Supplementary Figure 9

Isolated RNA does not contain DNA contamination. Omitting the genomic elimination step results in extracted RNA containing DNA (RNA/DNA sample). Primers were designed in two exons flanking introns to avoid possible amplification of genomic DNA. (**A**) PCR analysis of intronless U2 snRNA from an RNA sample without cDNA synthesis shows no DNA contamination. (**B**) RNA samples extracted from isolated nuclei (N) or cytoplasmic (C) fractions do not contain the Ptpn3 intron. (**C**) qRT-PCR using primers that flank a *Dazl* intron shows no DNA product. Primers (arrows) were designed according to a scheme reported below the image.

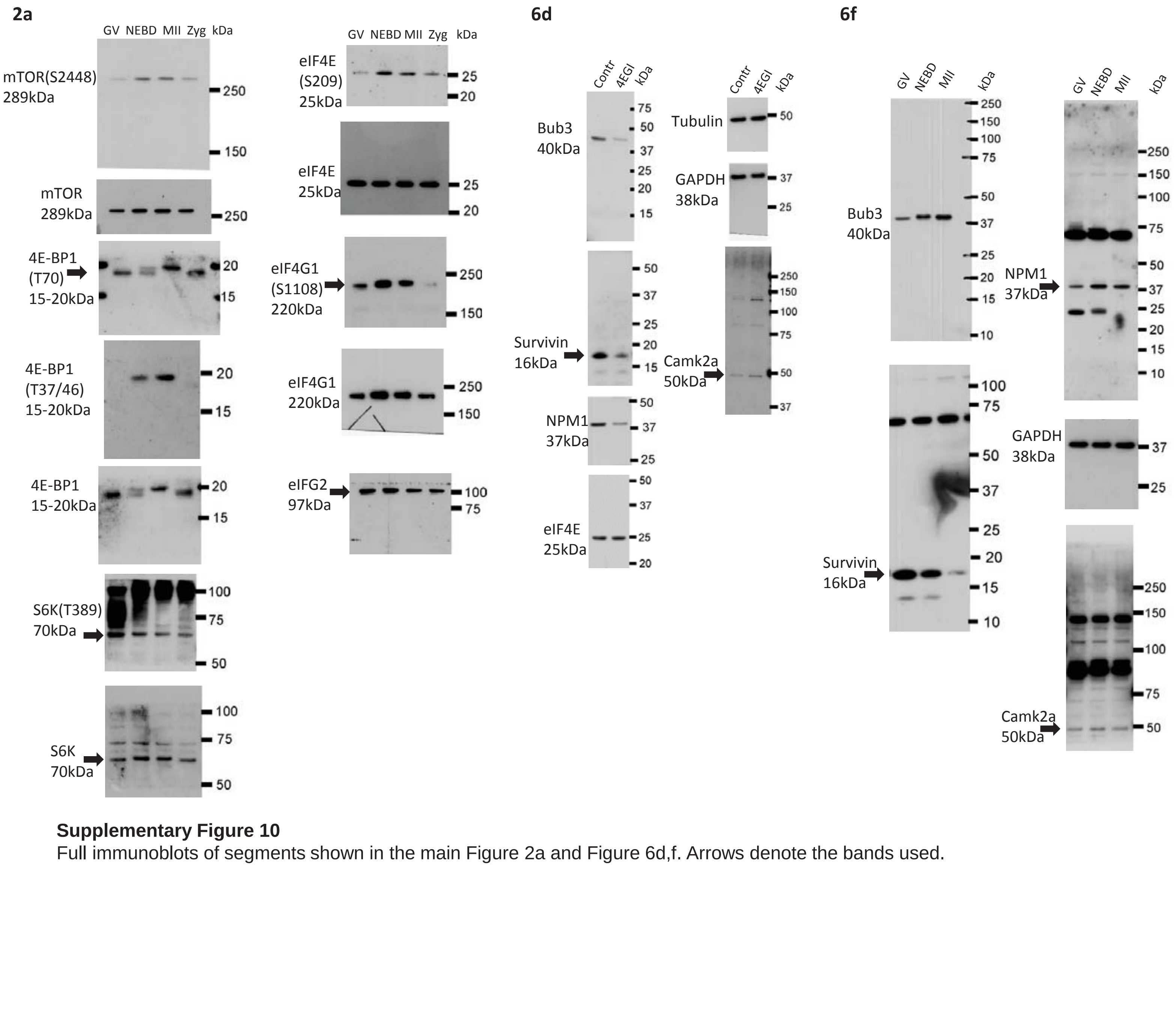

Supplementary Figure 10

Full immunoblots of segments shown in the main Figure 2a and Figure 6d,f. Arrows denote the bands used.

Supplementary Table 1
Primers used for PCR in this study.

Official symbol	Forward 5´-3´	Reverse 5´-3´	Gene Bank ID	Amplicon size (bp)	Annealing temperature (°C)
Birc5 / Survivin	GCTTCATCCACTGCCCTACC	TGCTCCTCTATCGGGTTGTCA	NM_001012273.1	106	58
Bub3	ACTCACGATGCCCCTATCAG	TTCTGGGGTCCCACAGTTTA	BC025089.1	100	58
CamK2a	TCCTGCCGCATTGAAGGAC	GAGAGCTGGCTGTTCACTGT	NM_009792.3	214	62
Dazl	GGATGAAACCGAAATCAGGA	ATAGCCCTTCGACACACCAG	NM_010021	94	58
Dazl E2/E3	GCAAAATCATGCCAAACACC	CAAATCCATAGCCCTTCGAC	NM_010021.5	150	50
Dazl E4/E5	GACGTGGATGTGCAGAAGATA	GGCTGCACATGATAAGTACA	NM_010021.5	110	50
Dazl E8/E9	ATACCTCCGGCTTATACAAC	AAGCTGGAGCAGCATCATG	NM_010021.5	103	50
Dazl E9/E10	CACTGCAGTGAAGTTGATC	CTTGAAGTAGTCATCTTGA	NM_010021.5	173	50
Gapdh	TGGAGAAACCTGCCAAGTATG	GGTCCTCAGTGTAGCCCAAG	XM_001476707.3	93	58
Mos	GTATAAAGCCACTTACCACGG	CAATGTTCAGTTCAGCCCA	NM_020021.2	106	60
mTor	GCCGGCCAGTCAGTAGAAAT	TCGTGAGATGTTGCCTGCTT	NM_020009.2	257	58
Neat2	AGGGAAAAGGGGGAAAGC	AGGGGTGAAGGGTCTGTGAT	NR_002847.2	133	58
Npm1	GCATGTCTGGAAAGCGATCT	TCATCGTCCTCATCATCGTC	NM_008722.3	100	58
Pabpn1l	TTCGCACAGATCTGTCAAG	TAGGGAGAGAACCACTGTG	NM_001007462.1	243	58
Ptpn3	GTGCAGGTTCCTCCGCCCAC	AGGGGCCCCACCGCATGTAT	NM_011207.2	884	58
Rnu 2-10	CAAGTGTAGTATCTGTTCTTATCA	GCTCCTATTCCAACTCCTA	NR_004414.1	101	60
Rnu12	ATAACGATTCGGGGTGACGCC	ATCCCGCAAAGTAGGCGGGTC	NR_004432.2	102	58
Tuba3a	AGGACCAGTGGTGAGGAACG	TTGCCGATCTGGACACCTG	NM_009446.2	110	58
